# Geospatial analysis of cholera patterns in Nigeria: findings from a cross-sectional study

**DOI:** 10.1186/s12879-021-05894-2

**Published:** 2021-02-23

**Authors:** Eunice Adeoti Salubi, Susan J. Elliott

**Affiliations:** Department of Geography and Environmental Management, University of Waterloo, 200 University Avenue West, Waterloo, ON N2L 3G1 Canada

**Keywords:** Cholera cluster, Local Moran’s I, Nigeria, Inland

## Abstract

**Background:**

Persistence of cholera outbreaks in developing countries calls for concern and more targeted intervention measures for long-term control. This research undertook spatial analysis of cholera incidence in Nigeria over a seventeen-year period to determine the existence of regional hotspots and predictors.

**Methods:**

A cross-sectional study design was used for the research. Cholera data for each of the thirty-six states and the federal capital territory (FCT) were obtained from the Nigeria Centre for Disease Control (NCDC) of the Federal Ministry of Health, Nigeria. Socioeconomic data including proportion of households using solid waste disposal (unapproved dumpsite, refuse burying, refuse burning, public dumpsite, and refuse collectors), water sources (pipe borne water, well, borehole, rain water, surface waters and water vendors), sewage disposal (water closet, pit latrines, bucket/pan, public toilet and nearby bush/stream), living in a single room and earning less than minimum wage (18,000 naira) were obtained from National Population Commission. On the other hand, proportion of illiterate adults (15 years and above) and poor people; and population density were obtained from National Bureau of Statistics. Each socioeconomic data was obtained at state level.

Cholera patterns were analysed at state level using Global Moran’s I while specific locations of cholera clusters were determined using Local Moran’s I. Stepwise multiple regression was used to determine socioeconomic predictors of cholera incidence.

**Results:**

Local Moran’s I revealed significant cluster patterns in 1999, 2001, 2002, 2009 and 2010 in Adamawa, Gombe, Katsina, Bauchi, Borno, Yobe, and Kano states. Households using surface water was the significant predictor (23%) of the observed spatial variations in cholera incidence.

**Conclusions:**

Persistence of cholera outbreaks in some north east and north western states calls for more targeted, long-term and effective intervention measures especially on provision of safe sources of water supply by government and other stakeholders.

## Background

Cholera is an acute diarrheal disease that continues to pose health challenges in developing countries [1]. Seven global cholera pandemics have been documented and the first pandemic originated from the Indian subcontinent and Southeast Asia in 1817 [2]. Two centuries after, an end is not in sight. As of 2021, the current pandemic has lasted six decades. Notable epidemics recorded in the last decade of the seventh pandemic include that in Yemen (over a million suspected cases) in 2017 with a case fatality rate (CFR) of 0.2% [3] and Haiti (340,311 cases; CFR of 0.84%) in 2011 [4].

Since its introduction into the African continent in 1970, cholera surges are still being experienced [5]. For instance, Somalia, Democratic Republic of Congo (DRC), South Sudan and Nigeria led the continent in 2017 [3]. In 2018, of the seventeen African countries that reported 120,652 cholera cases with a CFR of 2.0%, Nigeria took the lead recording about 37.3% of cases reported in the continent [1]. The recent 2018 cholera outbreak in Nigeria of over forty-five thousand cases and a CFR of 1.9% reflects an elevated burden of the disease in the country [6]. In addition, Nigeria was the only country that reported cholera in the West African sub region with an exception of Liberia that reported only two cases [1] and is among the forty-seven countries targeted for cholera control by 2030 [7].

The pandemic nature of cholera reflects the appearance and reappearance of *Vibrio cholerae* (*V. cholerae*) at different periods. This may suggest the influence of both natural and anthropogenic factors. *V. cholerae* thrives best in brackish waters with increased aquatic plankton, moist environments, and warm temperatures. Hence, potential cholera outbreaks may result where favourable interactions exist between the causative infectious agent and the population.

While factors such as poor sanitation, open drainage conditions, and contaminated water sources symbolize cholera endemic areas in coastal areas [8, 9], spatiotemporal heterogeneities of inland cholera outbreaks are not well explored. In a bid to contribute to the mandate of the global health agenda of 2030, this study seeks to provide information that will broaden the range of options for policy modification through its investigation of how spatiotemporal variations in inland regions influence cholera outbreaks.

## Methods

### Study area

**Fig. 1:**
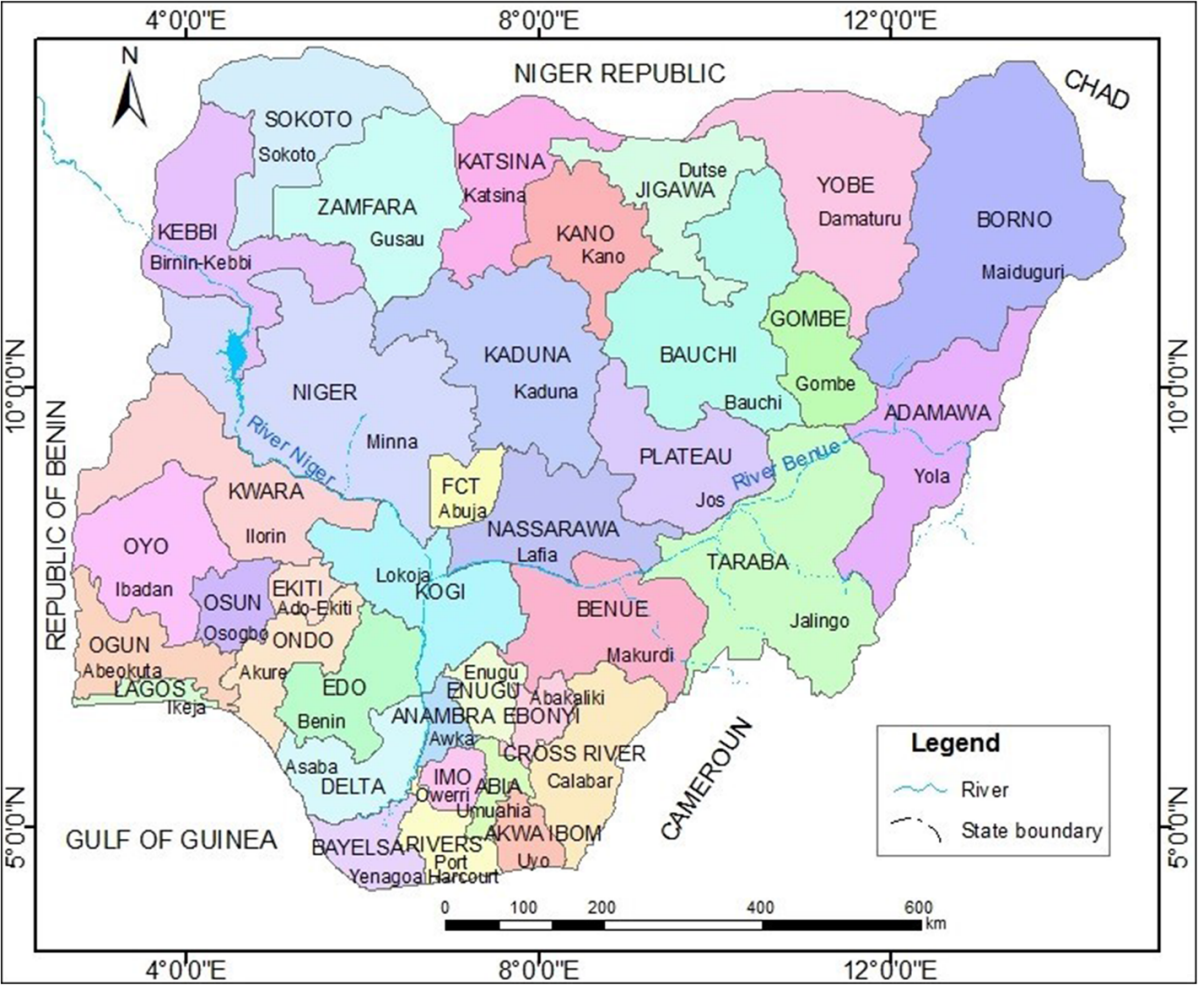
Nigeria showing the thirty-six states and the Federal Capital Territory (FCT). Map generated using ArcGIS version 10.6 software

Nigeria lies in tropical West Africa between latitudes 4^0^ and 14^0^ North of the equator and longitudes 3^0^ and 15^0^ East of the Greenwich Meridian (Fig. 1). It has a total surface area of 923,768 km^2^ comprising land and water area of 910,768 km^2^ and 13,000 km^2^, respectively. It is about four times the area of Ghana and about thirteen times the area of Sierra Leone. Its administrative units comprise 36 states and the Federal Capital Territory (FCT) in Abuja as well as 774 Local Government Areas. The choice of Nigeria for the study is based on the fact that waterborne and water-related diseases such as cholera and diarrhoea are still endemic [6, 10]. In addition, Nigeria is an interesting region to explore as there are coastal and landlocked areas, temperature variations, species variety in vegetation/trees, relief variations and seasonal changes. Factors such as relief, climate, seasons and water resources may play vital roles in potential cholera outbreaks in the country.

Nigeria’s relief can be divided into highlands and lowlands, and further subdivided into several land units. Generally, altitudes of highlands range between 600 and 2000 m subdivided into three: the North Central Plateau, the Eastern and North-eastern highlands, and the Western Uplands. On the other hand, lowlands, usually less than 150 m, can be subdivided into five land units: the Sokoto Plains, the Niger/Benue Trough, the Chad Basin, the interior coastal lowlands of western Nigeria, and the coastlands. Such relief features may influence cholera outbreaks.

Climatic factors such as temperature, rainfall and seasons have also been known to be associated with cholera outbreaks. Temperatures are generally high throughout the year because Nigeria lies within the tropics and close to the equator. Rainfall is a major climatic variable that differentiates wet and dry seasons. Similar to temperature, rainfall decreases both in duration and amount from over four thousand millimetres spread over eight to ten months along the coast to less than two hundred and fifty millimetres in the interior (extreme north) spread over three to four months. However, altitudinal effects create islands of greater rainfall in highlands such as the Jos Plateau. A single peak regime and a double peak regime characterise the north and south, respectively. These climatic factors may influence spatial and temporal variations of cholera.

Quality of water supplies in a community can be related to health conditions. Nigeria is naturally endowed with an uneven spread of abundant surface and groundwater resources but they are poorly managed and harnessed [11] leading to water scarcity especially in the north. Coupling with climate change, significant increases in evapotranspiration with lowering water levels during prolonged dry seasons in the north are experienced. On the other hand, climate change aids increase in coastal floods and saline water intrusion in the south or tropical rainforest zone. Population growth will continue to exert pressure on surface and ground water with subsequent intrusions of pollutants (chemical and biological industrial discharges or household/human wastes) into water courses that may be hazardous to health.

### Study design

A cross-sectional study design was used for the research because the study attempted an analysis of the relationship between predicted (cholera) and predictor (socioeconomic) variables at a specified period. Secondary data were obtained and used for the research.

### Cholera and population data

Cholera morbidity and mortality data for each of the thirty-six states and the FCT from 1997 to 2013 were obtained from the Nigeria Centre for Disease Control (NCDC) of the Federal Ministry of Health, Abuja. According to NCDC, reported cholera cases were based on the World Health Organisation (WHO) standard case definition: patients five years of age or older with acute watery diarrhoea, with or without vomiting. Population data for each state and the FCT were obtained from 1991 and 2006 national population censuses conducted by National Population Commission (NPC). Population was projected for the years with unavailable population census figures. For 2007 to 2013, population data for the thirty-six states and the FCT was projected at 3.2% growth rate of Nigeria (NPC, 2006) from 2006 population census figures; while population data for 1997 to 2005 was projected at 2.8% growth rate from 1991 population census figures. Cholera incidence rate for a state was expressed as a ratio of cholera morbidity cases in a state to its total population per 100,000 people.

Temporal data were mined from WHO publications using yearly cholera data series from 1997 to 2019. However, cholera data from 2014 to 2019 were unavailable at state level.

### Socioeconomic data

Socioeconomic data were obtained from publications of NPC and National Bureau of Statistics (NBS). Proportion of households using solid waste disposal (unapproved dumpsite, refuse burying, refuse burning, public dumpsite, and refuse collectors), water sources (pipe borne water, well, borehole, rain water, surface waters and water vendors), sewage disposal (water closet, pit latrines, bucket/pan, public toilet and nearby bush/stream), living in a single room and earning less than minimum wage (18,000 naira) at state level were obtained from NPC for 2006. Proportion of illiterate adults (15 years and above) and poor people; and population density at state level were obtained from NBS for 2008. Socioeconomic data were projected to 2013 at 3.2% growth rate.

### Data analysis

A composite map showing the spatial pattern of cholera incidences from 1997 to 2013 was generated in ArcGIS 10.6 (ESRI Inc.). The composite map was produced by finding averages of incidences in each state and the FCT over the seventeen-year period. Spatial autocorrelation tests were performed using Global Moran’s I and Local Moran’s I on ArcGIS software to reveal patterns of cholera distribution and localised clusters, respectively. It should be noted that according to ESRI’s documentation, a minimum of thirty spatial features is required for Moran’s I analysis on ArcGIS. So, this study based on thirty-seven administrative units is fairly above the requirement.

Spatial autocorrelation measures determine correlation within variables across a georeferenced space. Moran’s I is a common and widely used measure and test for spatial autocorrelation which involves only one variable such as crime, mortality, or incidence and can be used for both point and polygon analysis. Moran’s I values range on a scale between − 1 (high negative spatial autocorrelation or perfect dispersion) through 0 (no spatial autocorrelation or random spatial pattern) to + 1 (high positive spatial autocorrelation or perfect correlation). For statistical hypothesis testing, Moran’s I values can be transformed to z-scores in which values greater than 1.96 or smaller than − 1.96 indicates spatial autocorrelation, at 5% significance level.

However, Moran’s I statistic cannot distinguish between a hotspot (a place where high values cluster together) or a cold spot (a place where low values cluster together) which led to the development of local spatial autocorrelation techniques such as Local Moran’s I, a statistic which identifies localised clusters of features with values similar in magnitude as well as outliers by comparison to neighbouring features and the mean of the entire population. Spatial clusters are represented as either high-high (high values surrounded by high values) or low-low (low values surrounded by low values). Spatial outliers are represented as either high-low (high values surrounded by low values) or low-high (low values surrounded by high values).

A stepwise multiple regression was performed on SPSS IBM 20 to determine best predictors for cholera variance. In stepwise regression, statistically significant predictor variables are entered into the regression equation. A preliminary correlation analysis is essential to examine highly correlated variables that need to be excluded to achieve an assumption of a multiple linear regression framework.

In essence, a preliminary correlation analysis of the twenty-one variables was done and variables that were highly correlated (> 0.70) were excluded. As a result, five variables were excluded: households using water closet, refuse collection, and unapproved dumpsites; households living in single rooms and population density. A stepwise multiple regression was then performed on sixteen variables to determine the best predictors for cholera. However, due to the nature of stepwise regression which takes advantage of chance, it is important to conduct a cross-validation exercise. Therefore, an initial stepwise regression was performed before an independent validation to ascertain the validity of the model.

An initial stepwise regression was run on all samples using sixteen variables. Afterwards, an independent validation was done by taking a random sample of 75% of all cases and running the stepwise regression again using similar variables. The model is validated when the same variables that loaded on our full sample also load on our independent or training sample. In addition, the model is valid when the amount of variance (r^2^) is equivalent for our full sample as it was for our training sample. Cholera and socioeconomic data were log-transformed to normalize the data before running the regression analysis. Analyses were carried out at *p* ≤ 0.05 level.

Annual cholera cases from 1997 to 2019 was plotted to reflect trends of cholera in Nigeria. Time was plotted on the x-axis, while annual cholera data was plotted on the y-axis. A temporal correlation analysis was done to evaluate the randomness in the data series using correlograms. Correlograms are autocorrelation plots that display temporal correlation of a single time series variable. The autocorrelation plots come in two forms: autocorrelation function (ACF) and partial autocorrelation function (PACF). The ACF measures and plots average correlation between cholera data at a current period with data at previous time periods for different lag lengths. The PACF measures the correlation between cholera data at two time periods provided they are correlated to data at other time periods.

## Results

**Fig. 2:**
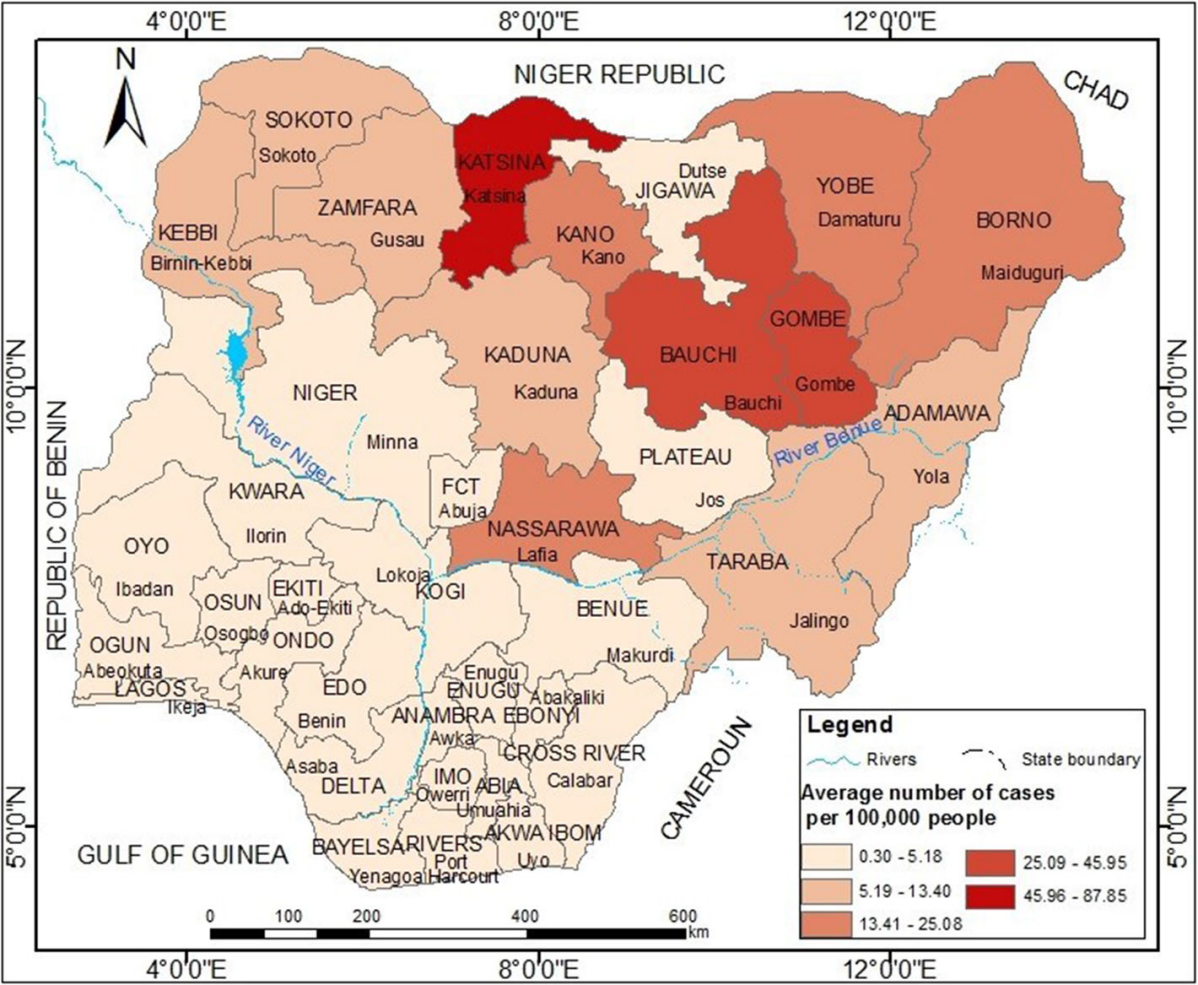
Spatial pattern of cholera incidence (1997–2013). Map generated using ArcGIS version 10.6 software

A total of 256,228 cases and 9061 deaths were reported between 1997 and 2013 with a CFR of 3.5%. The composite map (Fig. 2) reveals that all states in the northwest and the northeast except Jigawa recorded high incidences. The highest incidences were recorded in Katsina (87.85 cases per 100,000) followed by Gombe (45.95 cases per 100,000) and Bauchi (32.95 cases per 100,000). On the other hand, all states in the southwest, south and southeast regions recorded the lowest incidences of less than 5.2 cases per 100,000. The FCT and all states, excluding Nassarawa, in the central parts of the north also recorded less than 5 cases per 100,000. Jigawa was the only state in the northeast with the least incidence of approximately 3 cases per 100,000.

Global Moran’s I statistic was employed to determine patterns of distribution of cholera incidences from 1997 to 2013 (Table 1). Table 1 comprises Moran’s I, z-score, *p*-value and pattern for each year. Spatial random patterns were reflected in most of the years (1997, 1998, 2000, 2003–2008, and 2011–2013). However, 1999, 2001, 2002, 2009 and 2010 reflected statistically significant spatial cluster patterns.

**Table 1:** Global Moran’s I Statistic

Year	Moran’s I value	Z-score	p-value	Pattern
1997	0.06216	0.938323	0.348078	Random
1998	0.08851	1.346367	0.178184	Random
1999	0.2834	3.428076	0.000608	Cluster
2000	0.06789	0.990424	0.321967	Random
2001	0.20931	3.117124	0.001826	Cluster
2002	0.22643	2.641001	0.008266	Cluster
2003	0.00365	0.389753	0.696719	Random
2004	−0.0079	0.422054	0.672986	Random
2005	0.00941	0.574035	0.565944	Random
2006	0.00215	0.523569	0.600579	Random
2007	0.018	0.741835	0.458187	Random
2008	0.01057	0.752317	0.45186	Random
2009	0.15669	2.271743	0.023102	Cluster
2010	0.30816	3.648136	0.000264	Cluster
2011	0.11739	1.603117	0.108909	Random
2012	0.0187	0.541518	0.588151	Random
2013	0.03583	0.934177	0.350213	Random

**Fig. 3:**
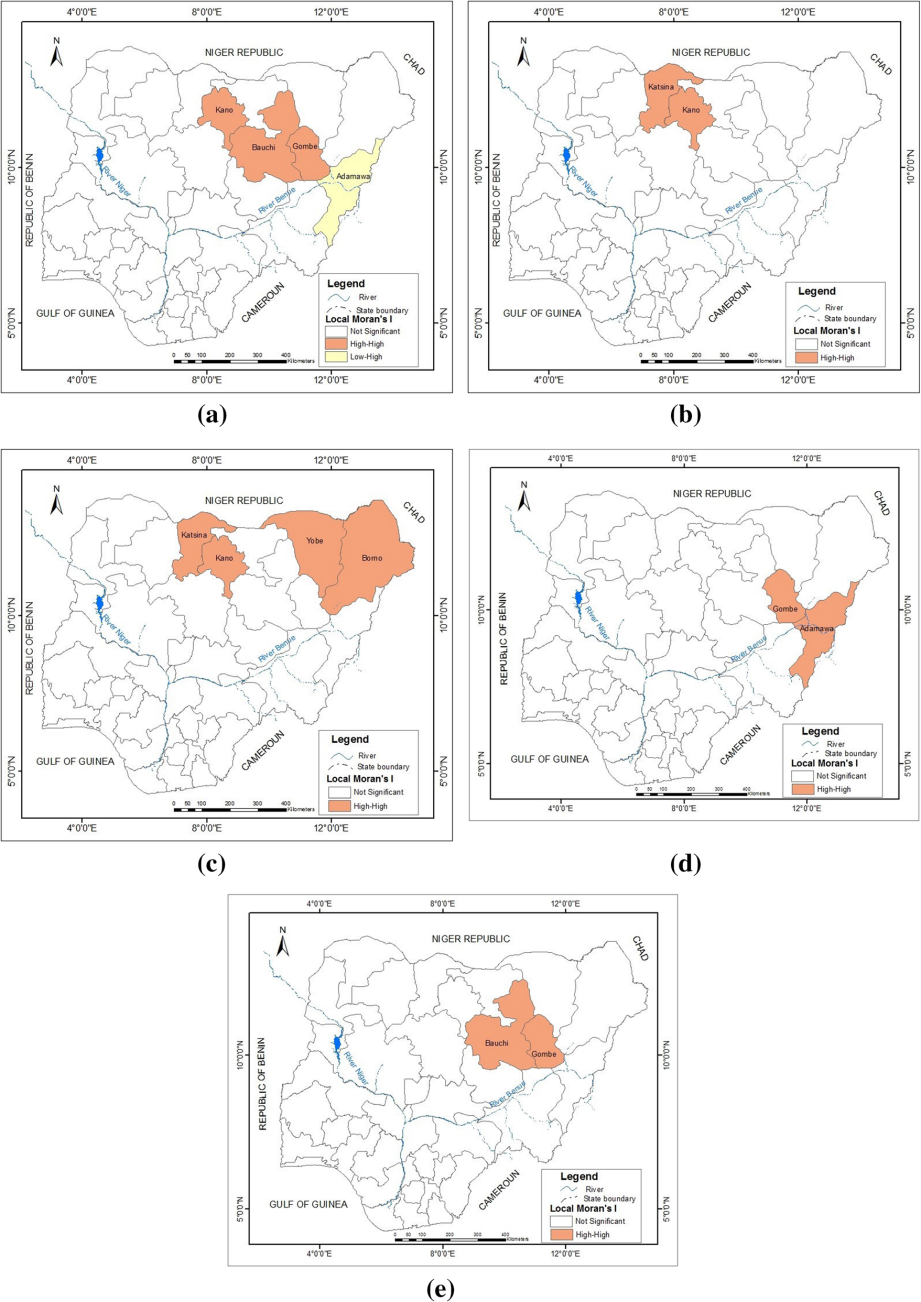
Spatial cluster of cholera incidence (**a**) 1999 (**b**) 2001 (**c**) 2002 (**d**) 2009 (**e**) 2010. Map generated using ArcGIS version 10.6 software

Anselin’s local Moran’s I was further employed to reveal specific areas of significant spatial clusters (Fig. 3). 1999 revealed concentrations of spatial clusters (high-high) in Kano, Bauchi and Gombe states while Adamawa state represented a spatial outlier (low-high). All other states including the FCT were not statistically significant. By 2001, only Katsina and Kano states revealed significant spatial clusters (high-high). Similarly, in 2002, only Katsina, Kano, Yobe and Borno states had spatial clusters (high-high). By 2009, a shift in spatial clusters to the north east was observed as reflected in Gombe and Adamawa while this continued till 2010 with a spatial cluster (high-high) in Bauchi and Gombe states. These results show that spatial clusters were concentrated in only a few states while most states were not statistically significant. High-high regions represent high-incidence states bordered by high-incidence state(s) while a low-high region is a low-incidence state bordered by a high-incidence state.

Tables 2 and 3 show the result of the initial stepwise regression analysis. The multiple correlation coefficient, r, was 0.48 while r-squared was 0.23, indicating approximately 23% of cholera variance was accounted for by surface water. ‘Surface water’ was the only statistically significant predictor that entered the regression equation. Results of the independent validation (Tables 4 and 5) revealed ‘surface water’ as the only predictor that entered the regression equation while r and r^2^ were approximately 0.47 and 0.22, respectively. Therefore, a similarity was observed in the resulting predictor: surface water; and the amount of variance in the full sample was equivalent to that of the training sample using 75% of all cases. This validates the model. However, caution should be taken in interpreting the results as 23% in the r-squared of the predictor shows a fairly moderate contribution of surface water usage to cholera variance and that may suggest the possibility of other predictors that can be included in the model to make it more robust.

**Table 2:** Predictor of cholera using full sample

Model	Variables Entered	Variables Removed	Method
1	Surface water	.	Stepwise (Criteria: Probability-of-F-to-enter <= .050, Probability-of-F-to-remove > = .100).

Dependent Variable: Cholera cases

**Table 3:** Regression model summary^b^ using full sample

Model	R	R Square	Adjusted R Square	Std. Error of the Estimate	Change Statistics				
					R Square Change	F Change	df1	df2	Sig. F Change
1	.480^a^	.230	.208	.99090	.230	10.476	1	35	.003

a. Predictors: (Constant), Surface water

b. Dependent Variable: Cholera cases

**Table 4:** Predictor of cholera using training sample

Model	Variables Entered	Variables Removed	Method
1	Surface water	.	Stepwise (Criteria: Probability-of-F-to-enter <= .050, Probability-of-F-to-remove > = .100).

a. Dependent Variable: Cholera cases

b. Models are based only on cases for which Approximately 75% of the cases (SAMPLE) = 1

**Table 5:** Regression model summary^b,c^ using training sample

Model	R	R Square	Adjusted R Square	Std. Error of the Estimate	Change Statistics				
	Approximately 75% of the cases (SAMPLE) = 1 (Selected)				R Square Change	F Change	df1	df2	Sig. F Change
1	.467^a^	.218	.194	1.01822	.218	8.928	1	32	.005

a. Predictors: (Constant), Surface water

b. Unless noted otherwise, statistics are based only on cases for which Approximately 75% of the cases (SAMPLE) = 1.

c. Dependent Variable: Cholera cases

**Fig. 4:**
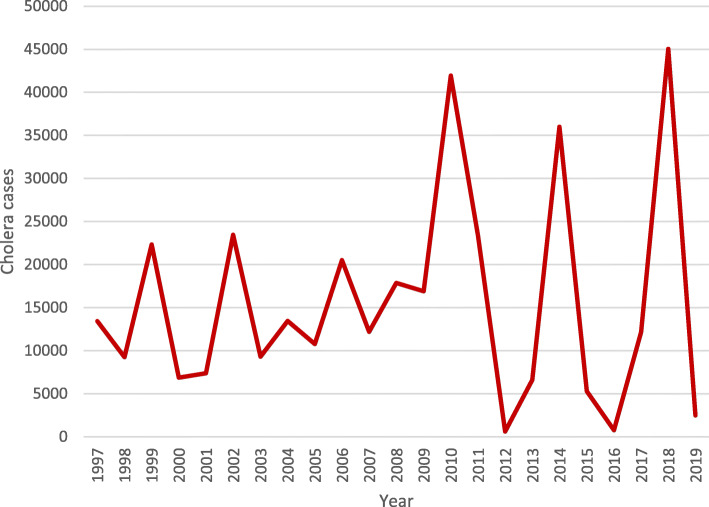
Annual cholera cases in Nigeria (1997–2019)

**Fig. 5:**
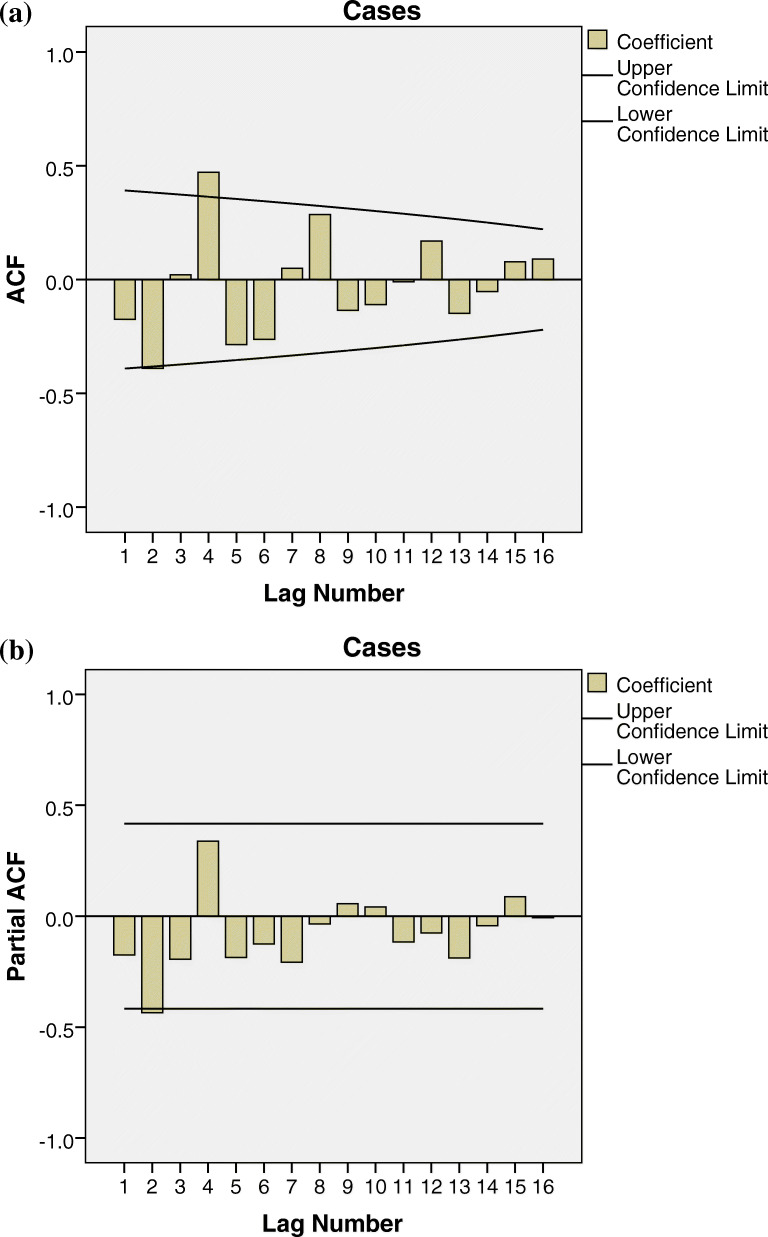
Temporal correlation using correlogram (**a**) autocorrelation function plot (**b**) partial autocorrelation function plot

Figure 4 reveals a striking pattern of three major cholera peaks in 2010 (41,943 cases), 2014 (35,996 cases) and 2018 (45,037 cases) and three minor peaks in 1999, 2002 and 2006. From the temporal pattern, a four-year cycle is observed between the three major peaks. Temporal correlation analysis using correlograms reveals ACF and PACF values at each period (Fig. 5), while the two horizontal lines represent significant thresholds. Only the vertical lines that exceed the two horizontal lines are considered significant. Only one ACF value is therefore significant (Fig. 5a). Other ACF and PACF values are not statistically significant; the correlogram thus shows a non-random data set.

## Discussion

High incidences of cholera recorded in the north western and north eastern states over the seventeen-year study period reveal a striking pattern of cholera predominance there. Due to unavailable data on smaller administrative units within each state across the country, identification of localities where high incidences were prevalent was not possible. Further studies are thus needed in analysing cholera incidences at the local government or ward level to provide information for more targeted intervention measures. Global Moran’s I revealed statistically significant spatial cluster patterns in 1999, 2001, 2002, 2009 and 2010, while Local Moran’s I revealed concentrations of spatial clusters for those years. Local Moran’s I analysis revealed concentration of spatial clusters (high-high) in only a few northern states: Kano, Bauchi and Gombe in 1999; Katsina and Kano in 2001; Katsina, Kano, Yobe and Borno in 2002; Gombe and Adamawa in 2009; and Bauchi and Gombe in 2010. Only in 1999 was there a spatial outlier (low-high) in Adamawa state. All other states including the FCT were not statistically significant. This finding reinforces on states where high cholera incidences are prevalent. However, the results should be taken with caution because according to ESRI’s documentation, results of Moran’s I analysis can be reliable only when the input features are a minimum of 30 features; although this analysis involved 37 features, it is fairly above the required minimum which may account for only the spatial clusters in a few states and the non-statistically significant features being recorded in most of the other states. More rigorous analyses at smaller administrative units are therefore needed to reinforce on the reliability and robustness of the result.

Findings from the stepwise multiple regression revealed households using surface water as the only significant predictor with about 23% of cholera variance being attributed to it. A cross-validation exercise of the model via an independent sample proved its validity. Households using surface water was defined as a proportion of population obtaining their water supply from ponds, rivers and lakes. This finding suggests a link in cholera dominance in the north western and north eastern states to the use of surface water that may be regularly exposed to contamination posing increased cholera risk to residents. Contamination may be introduced into surface water via pollutants from nearby sewage/waste disposal [8, 12–16]. There is the possibility of residents in the north subscribing more to the use of surface water for drinking and other domestic purposes due to regular groundwater or tap water shortages. Groundwater levels in the north are usually low and are poorly harnessed and managed; hence such water resources get depleted easily. Poor management of tap water by relevant water authorities at the local and state levels may also force residents to make use of surface water for their daily needs. Although this model was validated, the amount of variance suggests the evaluation of other predictors. A limitation of this study was that environmental predictors such as temperature and rainfall were not included in the regression analysis because such data were unavailable for all states, including the FCT for the period of study. Therefore, to address this limitation, follow-up studies that will include additional predictors are required.

Major and minor cholera peaks revealed from the temporal analysis suggest a lack of continuity in intervention measures such as safe water provision. It appears governments and other stakeholders seem to respond to provision of appropriate intervention measures only after major outbreaks of cholera in the country. So once a decline is being observed in the disease trend, provision of safe water sources begin to dwindle forcing the population to resort to unsafe water sources which then lead to recurrences of further outbreaks. Therefore, there is the need for consistent provision of safe water to residents across the country, and especially to those in the north east and north west to prevent further cholera outbreaks. A limitation of this study was that monthly cholera data were unavailable. Further studies are thus needed on monthly analysis to reveal more underlying factors responsible for the prevailing trend.

## Conclusions

Cholera cluster analyses are essential in providing localized regions of cholera intensity that will allow for targeted intervention measures [17] and provide a significant step toward cholera control while achieving the global mandate of the Sustainable Development Goals (SDGs) [7]. Government and other stakeholders should channel more resources towards provision of safe water especially in the north east and north western parts of the country. There is the need for more rigorous analytical studies that address the limitations of the current study.

## Data Availability

The datasets used and/or analysed during the current study are available from the corresponding author on reasonable request.

## Abbreviations

CFR: Case Fatality Rate
DRC: Democratic Republic of Congo
FCT: Federal Capital Territory
NCDC: Nigeria Centre for Disease Control
WHO: World Health Organisation
NPC: National Population Commission
NBS: National Bureau of Statistics
SDGs: Sustainable Development Goals
ACF: Autocorrelation function
PACF: Partial Autocorrelation function
